# Effect of *Schistosoma mansoni* infection and its treatment on antibody responses to measles catch-up immunisation in pre-school children: A randomised trial

**DOI:** 10.1371/journal.pntd.0007157

**Published:** 2019-02-14

**Authors:** Robert Tweyongyere, Beatrice R. Nassanga, Allan Muhwezi, Matthew Odongo, Swaib A. Lule, Rebecca N. Nsubuga, Emily L. Webb, Stephen C. Cose, Alison M. Elliott

**Affiliations:** 1 Department of Veterinary Pharmacy Clinical and Comparative Medicine, Makerere University, Kampala, Uganda; 2 Medical Research Council/Uganda Virus Research Institute and London School of Hygiene & Tropical Medicine Uganda Research Unit, Entebbe, Uganda; 3 London School of Hygiene & Tropical Medicine, Keppel Street, London United Kingdom; Centers for Disease Control and Prevention, UNITED STATES

## Abstract

**Background:**

*Schistosoma* infection is associated with immune modulation that can influence responses to non-schistosome antigens. Vaccine responses may be impaired in *S*. *mansoni*-infected individuals. We investigated effects of *S*. *mansoni* infection on responses to childhood measles catch-up immunisation and of praziquantel treatment on this outcome in a randomised trial.

**Methodology:**

The Immune Modulation and Childhood Immunisation (IMoChI) study was based in Entebbe, Uganda. Children aged 3–5 years (193 *S*. *mansoni*-infected and 61 uninfected) were enrolled. Infected children were randomised in a 1:1:1 ratio to receive praziquantel 2 weeks before, at time of, or 1 week after, measles catch-up immunisation. Plasma anti-measles IgG was measured at enrolment, 1 week and 24 weeks after measles immunisation. Primary outcomes were IgG levels and percentage of participants with levels considered protective against measles.

**Results:**

Anti-measles IgG levels increased following immunisation, but at 1 week post-immunisation *S*. *mansoni*-infected, compared to uninfected, children had lower levels of anti-measles IgG (adjusted geometric mean ratio (aGMR) 0.4 [95% CI 0.2–0.7]) and the percentage with protective antibody levels was also lower (adjusted odds ratio 0.1 [0–0.9]). Among *S*. *mansoni*-infected children, anti-measles IgG one week post-immunisation was higher among those treated with praziquantel than among those who were not yet treated (treatment before immunisation, aGMR 2.3 [1.5–4.8]; treatment at immunisation aGMR 1.8 [1.1–3.5]). At 24 weeks post-immunisation, IgG levels did not differ between the trial groups, but tended to be lower among previously-infected children who were still *S mansoni* stool-positive than among those who became stool-negative.

**Conclusions and significance:**

Our findings suggest that *S*. *mansoni* infection among pre-school children is associated with a reduced antibody response to catch-up measles immunisation, and that praziquantel treatment improves the response. *S*. *mansoni* infection may contribute to impaired vaccine responses in endemic populations; effective schistosomiasis control may be beneficial for vaccine efficacy. This should be further explored.

**Trial registration:**

ISRCTN87107592.

## Author summary

Infection with the blood fluke *Schistosoma mansoni*, which causes Bilharzia, is common among communities living along the shores of Lake Victoria in Uganda and elsewhere around fresh water bodies in Africa. Infection with this parasite is known to alter the body’s immune responses, even to stimuli which are not directly related to the parasite. This alteration may have an adverse impact on responses to vaccines. However, there is still insufficient information on such effects and on whether treatment of schistosomiasis can reverse them. We conducted a study on the Entebbe peninsula in Lake Victoria to examine the effects of infection with *S*. *mansoni* parasites on responses to childhood measles catch-up immunisation, and the effect of treating the parasites on the responses. A cohort of 3–5 year-old children, either infected with *S*. *mansoni* or not, was enrolled and given measles immunisation. The infected children were treated for *S*. *mansoni* either before, at the time of, or a week after the immunisation. Levels of antibodies against measles were measured at one week and 24 weeks after immunisation as indicator of possible protection against measles. The levels of antibodies against measles were lower among the *S mansoni*-infected children than the uninfected children, and treatment improved this situation. This implies that *S*. *mansoni* infection is associated with reduced measles immunisation responses and that treatment improves the response. At 24 weeks post-immunisation, when all children had been treated for *S*. *mansoni*, at least 95% of children in all groups had protective levels of antibody. Considering that pre-school children are vulnerable to Bilharzia, an effective schistosomiasis control programme that includes this age group may be beneficial for vaccine efficacy, an issue that needs further investigation.

## Introduction

There is a striking geographical pattern in the efficacy of vaccines, some being less efficacious in the tropics than in temperate regions. This has been particularly well documented with regard to immunisation with BCG [1]. There is also evidence that the response to other vaccines, including those against measles, polio and typhoid, is sub-optimal in tropical regions [2]. Among several hypotheses advanced to explain this phenomenon, one is that chronic, immunomodulating helminth infections, which are prevalent in the tropics, inhibit the development of Th-type 1 immune responses required for protective immunity to many viral or bacterial pathogens [3]. Despite considerable discussion of this hypothesis, studies to date have largely examined effects on vaccines which are first given to young infants (such as BCG [4–8], hepatitis B [9] and tetanus toxoid [6, 7, 10–13]), an age-group relatively unaffected by helminth infections.

Measles immunisation is among vaccines that may have impaired responses in tropical countries [2, 14]. It is usually first given at age nine months. In Entebbe, among infants immunised against measles at age nine months at Entebbe Hospital, only 75% had “protective” antibody levels at age one year [15]. In a study among household contacts of measles patients in Uganda, vaccine effectiveness was found to be only 74% [16]. Recent measles outbreaks in Uganda have been associated, similarly, with evidence of low effectiveness of single-dose measles vaccine (70–75%) [17, 18] compared with other settings (median 84% for immunisation at 9 months, 92.5% for immunisation at 12 months) [19]. Catch-up and booster immunisation on “child days” is offered in Uganda to address this problem. Low vaccine titres and poor vaccine handling have been suggested as explanations for the low effectiveness. The possibility of a role of chronic helminth infection has not been widely explored [14].

*Schistosoma mansoni* infection is endemic in Uganda affecting mainly fishing communities [20] including children under five years old [21–23]. *S*. *mansoni* infection is associated with strong immunoregulation [24] and this has been shown to extend to unrelated antigens [25]. This might influence the responses to measles immunisations as a result of suppressed responses due to impaired or altered antigen presentation [26], or a Th-type 2 biased response [27, 28]. We hypothesised that immunogenicity to the measles vaccine is impaired in *S*. *mansoni* infected children and that praziquantel treatment of *S*. *mansoni* infected children will improve their responses to measles immunisation. Here we report findings of a study on the association between *S*. *mansoni* infection and antibody responses to catch-up measles immunisation among pre-school children, and of a randomised trial of the effect of praziquantel treatment on the measles vaccine response–the first trial of this kind.

## Methods

### Ethics statement

Ethical approval of the study was obtained from the Uganda Virus Research Institute Science and Ethics committee and the Uganda National Council for Science and Technology (HS 1307). Informed written consent was obtained from the parent or guardian of the children.

### Study setting and enrolment of participants

We conducted an observational study combined with an individually randomised, non-blinded, trial of praziquantel treatment for *S*. *mansoni* infection, investigating effects on the response to measles immunisation (the “Immune Modulation and Childhood Immunisation” study [IMoCh; registration number: ISRCTN87107592]). The study was based at the Uganda Virus Research Institute, Entebbe, Uganda between February 2013 and March 2015 among five fishing communities (Kigungu, Kasenyi, Rwanjaba, Bugonga and Nakiwogo) on the Entebbe peninsula of Lake Victoria. Children, three to five years old, were screened for *S*. *mansoni* infection, using the Kato-Katz technique [29] and a single stool sample. The screening was done in collaboration with the Vector Control Division of the Ministry of Health, Uganda. Children who were stool positive for *S*. *mansoni* were invited to participate in the study after obtaining written informed consent from their parents or guardians.

To obtain an *S*. *mansoni* uninfected comparison group, 85 children were randomly selected from among stool negative children and also enrolled after obtaining written informed consent from their parents or guardians. For this group, two additional stool samples were requested on two consecutive days. Those found negative on all three stool samples were further tested with *S mansoni* stool PCR, as previously described [30]. In brief, DNA from stored stool was extracted using a QIAamp DNA Mini Kit (Qiagen), purified in QIAamp spin columns, quantified on a Nano drop 2000c and diluted to 50ng/μl. Specific forward and reverse primers and Taqman probes were used in a multiplex, real-time PCR. *S*. *mansoni* DNA was detected using Ssp48F, Ssp124R and Ssp78T-TR with Texas Red and (BHQ2). Serial dilutions of a positive pool were included to set a ct value cut off for the test samples. DNA amplification, detection and data analysis were attained with the BIORAD CFX96 Real time system and Bio-Rad CFX manager. Children found to be negative on all three stool samples and on PCR were considered uninfected.

### Randomisation and measles immunisation

Children infected with *S*. *mansoni* and enrolled into the study were randomly assigned in a 1:1:1 ratio to three study groups. Group A received praziquantel treatment (PZQ) two weeks before measles immunisation, Group B received PZQ on the same day as measles immunisation, Group C received PZQ one week after measles immunisation (and after one-week blood samples had been taken). PZQ treatment against *S mansoni* was by single-dose at 40 mg/kg in accord with Ministry of Health guidelines. *S*. *mansoni* uninfected children (group D) received measles immunisation but no praziquantel treatment.

A randomisation code was generated using a program in Stata (Stata SE version 11 StataCorp, USA), by a statistician who did not participate in the clinical or laboratory work, and delivered to the field workers in sealed envelopes. The field team allocated randomisation numbers sequentially and treated participants according to the regimen indicated by the code. Praziquantel (Cipla Ltd, Pantalgaanga M.S India) was provided by the Vector Control Division.

Measles vaccine (1 ml single dose; Biofarma, Indonesia) was provided at Kigungu Health Centre located in the study area.

### Follow up of study participants and blood samples processing

Participants were followed-up at one week and 24 weeks after the measles immunisation. At enrolment and at both follow-up time points, blood samples were drawn into heparin for plasma. Plasma was stored at -80°C until needed for assaying of anti-measles antibodies. At the end of the study (24 weeks), children that still had evidence of patent worm infections were treated accordingly.

### Measurement of antibody responses to measles

Levels of measles-specific IgG in plasma were measured by the ELISA method, using a quantitative commercial kit (Enzygnost anti-measles IgG kit OWLN155 & OUVP175, Siemens, Germany) according to the manufacturer’s protocol and as previously described [15]. All three blood samples taken at the different time points from the same individual were assayed on the same plate; the sample sets from different individuals were randomised across the plates. The IgG levels were quantified in milli-International units per millilitre of plasma (mIU/mL) calculated based on the formula: log10 mIU/mL = α x Aβ (where α and β are lot-dependent constants provided by the manufacturer [31]). A protective response was defined as having a level of measles-specific IgG greater than 200 mIU/mL as described by Naniche [32].

### Statistical methods

The primary outcome measures considered in this analysis were levels of measles-specific IgG and percentage of children with IgG levels considered protective, measured at one week and at 24 weeks after measles immunisation.

Data analysis was performed with three main objectives: (1) at enrolment we assessed baseline measles-specific IgG responses and associations with measles immunisation history, *S*. *mansoni* infection, gender, and age; (2) at one week after measles immunisation we examined for effect of *S*. *mansoni* infection on antibody responses by comparing IgG levels and proportion with protective levels between stool negative children (group D) and *S mansoni* stool positive children who had not yet received praziquantel treatment (group C) in an observational analysis, adjusting for baseline IgG levels and prior measles immunisation history; (3) at both one week and 24 weeks after measles immunisation we examined for the effect of praziquantel treatment timing among *S*. *mansoni* infected children on responses to measles immunisation by comparing IgG levels between the three groups (A, B & C) who received praziquantel treatment at different times with respect to the immunisation. Antibody data was skewed and was log10 transformed. Unless otherwise stated, comparisons of antibody responses between and within study groups were done by logistic regression (for the binary outcome) or by linear regression (for the continuous outcome) with bootstrapping to generate 95% confidence intervals. Log10 transformed data were back-transformed to express differences between groups as geometric mean ratios.

## Results

### The study participants and baseline characteristics

**Fig 1:**
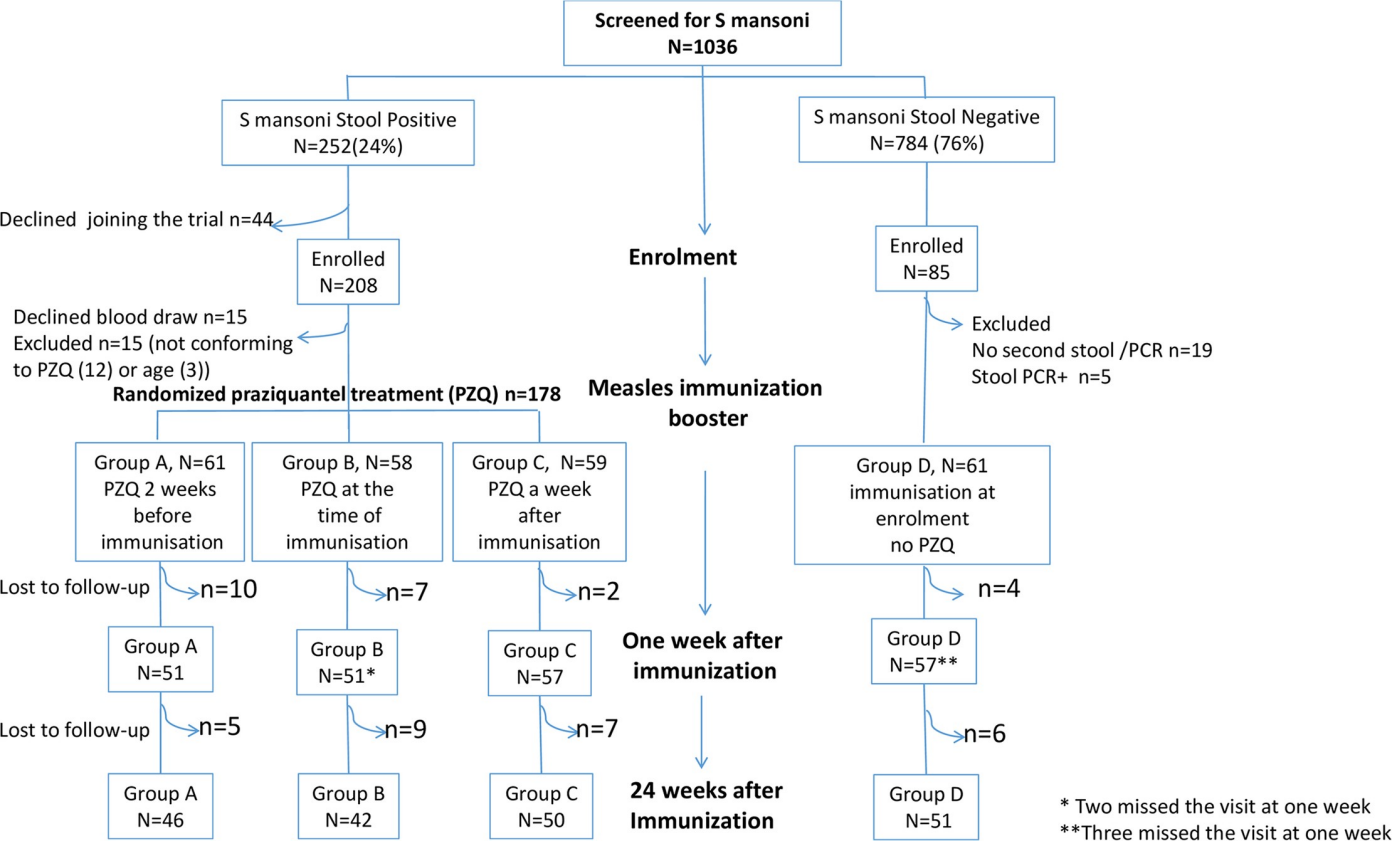
Flow diagram.

One thousand and thirty-six children aged three to five years were screened, of whom, 252 (24%) were positive for *S mansoni* based on a single stool sample. All *S*. *mansoni* infected children were invited to join the trial and 208 were enrolled but fifteen declined to have blood drawn while fifteen children were subsequently excluded either due to failure to conform to the treatment protocol (n = 12) or because they were later confirmed to be above the age of five (n = 3), leaving 178 children for randomisation to the study groups. From the comparison group of 85 stool negative children, 24 were excluded due to failure to provide additional stool samples (n = 19) or PCR positive stool (n = 5) (Fig 1).

Of the 239 participants, 96.2% reported not to have had any previous praziquantel treatment or other specific therapy against schistosomiasis. On the other hand, 172(72.0%) of the children were reported to have had measles immunisation at age nine months and 153 (64.0%) to have had measles immunisation during child health days’ campaigns in the past 12 months. Of note 124/239 (51.9%) reported to have had immunisation at both 9 months and during child health day campaigns in the past 12 months while 48/239 (20.1%) reported to have received immunisation at 9 months only, 29/239 (12.1%) only had immunisation during child health days and 38/239 (15.9%) reported to have never had any measles immunisation or were not aware of having any. However, only 9.6% children provided immunisation cards, in confirmation of the immunisation record. For the rest we relied on the report from the parent or guardian regarding immunisation history.

A summary of the baseline demographic characteristics of the study cohort is shown in Table 1. Among the *S*. *mansoni* infected children, characteristics were similar between the three randomised treatment groups except that group A (treated 2 weeks before immunisation) had somewhat lower pre-immunisation measles antibody levels.

**Table 1:** Baseline and demographic characteristics of the study cohort.

	*S mansoni* stool positive children			*S mansoni* stool negative children
	PZQ at two weeks before immunisation (Group A)	PZQ at Immunisation (Group B)	PZQ at a week after immunisation (Group C)	(Group D)
	N = 61	N = 58	N = 59	N = 61
**Gender**				
Males	25(41%)	35(60%)	30(51%)	23(38%)
Females	36(59%)	23(40%)	29(49%)	38(62%)
**Mean Age (years)**	3.8(SD = 1.1)	3.9(SD = 0.8)	4.1(SD = 1.0)	4.0(SD = 0.8)
**Other Worms**				
*Trichuris trichiura*	2(3.3%)	6(10.3%)	7(11.9%)	4(6.6%)
*Hymenolepis nana*	4(6.6%)	5(8.6%)	1(1.7%)	3(4.9%)
Ascaris	1(1.6%)	2(3.5%)	1(1.7%)	0
Hookworm	0	2(3.5%)	1(1.7%)	0
***S mansoni* intensity**				
Median epg (interquartile range)	132 (36, 396)	60 (24, 222)	48(24,244)	
WHO category				
Light	29(47.5%)	34(58.6%)	40(67.8%)	
Moderate	18(29.5%)	12(20.7%)	12(20.3%)	
Heavy	14(23.0%)	12(20.7%)	7(11.9%)	
**Reported previous schistosomiasis treatment**				
Yes	2(3.3%)	4(6.9%)	1(1.7%)	1(1.6%)
Never	58(96.7%)	54(93.1%)	56(96.6%)	60(98.4%)
Not sure	0	0	1(1.7%)	0
**Measles immunisation**				
At 9months				
Yes	49(80.3%)	41(70.7%)	46(78.0%)	36(59.0%)
No	9(14.8%)	7(12.1%)	4(6.8%)	7(11.5%)
Not sure	3(4.9%)	10(17.2%)	9(15.2%)	18(29.5%)
At child days in last 12 months				
Yes	33(54.1%)	40(69.0%)	38(64.4%)	42(68.9%)
No	24(39.3%)	10(17.2%)	16(27.1%)	11(18.0%)
Not sure	4(6.6%)	8(13.8%)	5(8.5%)	8(13.1%)
**Anti-measles IgG at enrolment**				
Median IgG levels mIU/mL (IQR)	818 (394, 2840)	1208 (521, 3406)	1090(579, 3962)	1277(438, 3832)
% with protective antibody levels	52(85.3%)	50(86.2%)	51(87.9%)	53(86.9%)

### Baseline anti-measles IgG levels

At enrolment, we explored the baseline measles–specific IgG levels among the children and examined possible associations with gender, reported immunisation history and infection with *S*. *mansoni*. Overall, the geometric mean IgG level at enrolment was 1099 mIU/mL (95% CI 851, 1421) and 86.6% of the participants had antibody levels considered protective against measles infection. As shown in Table 2, children with a history of previous measles immunisation had somewhat higher IgG levels (p = 0.05 for those immunised on child days). The baseline antibody levels showed a tendency to be lower with increasing *S*. *mansoni* infection intensity (p = 0.10). When adjusted for age, sex and the other variables in the model, the adjusted estimates were similar to crude estimates. We therefore adjusted for prior measles immunisation history and baseline measles antibody levels in subsequent analyses.

**Table 2:** Crude associations between participant characteristics and anti-measles IgG levels at enrolment.

	Geometric mean levels anti-measles IgG mUI/mL (95% CI)	Geometric mean ratio (95%CI)		% with protective antibody levels	Odds Ratio (95% CI)	
**Gender**						
Boys (n = 113)	888 (617, 1276)	1	p = 0.11	84.10%	1	p = 0.29
Girls(n = 125)	1334 (928, 1918)	1.5 (0.9, 2.6)		88.80%	1.5 (0.7, 3.2)	
**Measles immunisation history**						
At 9 months ^b^						
No^c^(n = 26)	1065 (423, 2685)	1	p = 0.94	80.80%	1	p = 0.28
Yes (n = 172)	1105 (827, 1478)	1.0 (0.4, 2.8)		88.40%	1.8 (0.6, 5.3)	
Child days ^b^						
No^c^ (n = 61)	721 (402, 1293)	**1**	**p = 0.05**	83.60%	1	p = 0.19
Yes(n = 152)	1385 (1025, 1872)	**1.9 (1.0, 3.5)**		90.10%	1.8 (0.8, 4.2)	
***S*. *mansoni* stool result**						
Negative(n = 61)	1312 (801, 2150)	1	p = 0.43	86.90%	1	p = 0.93
Infected (n = 177)	1034 (764, 1399)	0.8 (0.5, 1.3)		86.40%	1.0 (0.4, 2.3);	
***S*. *mansoni* intensity**						
Negative(n = 61)	1312 (801, 2150)	1	p = 0.10^a^	86.90%	1	p = 0.60
Light (n = 103)	1189 (800, 1769)	0.9 (0.5, 1.7)		88.40%	1.1 (0.4, 3.0)	
Moderate(n = 41)	1076 (564, 2051)	0.8 (0.3, 1.6)		87.80%	1.1 (0.3, 3.6)	
Heavy (n = 33)	637 (309, 1313)	0.5 (0.2, 1.0)			78.80%	0.6 (0.2, 1.7)

Baseline measles IgG was missing for one child.

^a^ Test for trend p value

^b^ Parents/guardians were not sure of measles immunisation status at 9 months for 40 children, and on child days for 25 children.

^c^ Note that children who had not received measles immunisation at 9 months may have received it on child days (reported for 14/26 cases), and those who had not received on child days may have received at 9 months (reported for 39/61 cases)

### Association between *S mansoni* infection and anti-measles IgG response to catch-up immunisation

**Fig 2:**
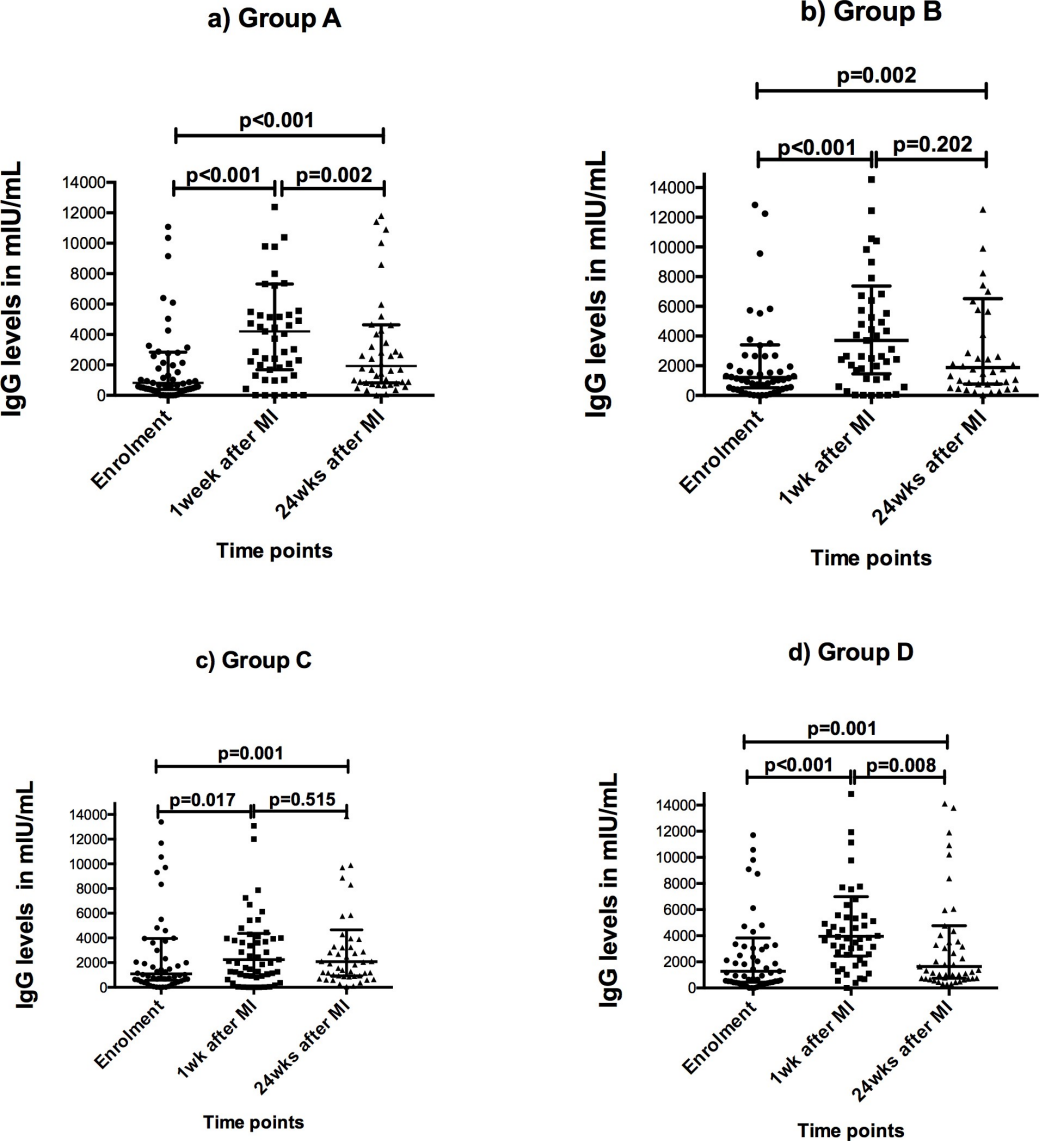
Anti-measles IgG levels at respective time points for each treatment group. Group A received praziquantel (PZQ) 2 weeks before measles immunization (MI), Group B received PZQ at the time of MI, Group C received PZQ one week after MI and Group D was *S mansoni* negative and did not receive PZQ. Shown on the dot plots are medians with inter-quartile whiskers and the Wilcoxon Signed-rank paired sample test p-values.

Overall, and as expected, there was a significant boost in measles antibody levels following the catch-up measles dose and this was seen for all study groups (Fig 2). The proportion with IgG levels considered protective was 86.6%, 90.1% and 96.8% at enrolment, one week and 24 weeks after immunisation respectively. Levels at 24 weeks after immunisation remained higher than enrolment levels although among groups A and D, they had declined significantly, to levels lower than those at one week after immunisation.

Having observed a boost in antibody levels we wanted to examine whether *S mansoni* infection had an effect on the boost in antibody levels. To do this, we compared the anti-measles IgG levels at one week between *S*. *mansoni*-infected children before their treatment (group C) and uninfected children (group D). As shown in Table 3, one week post-immunisation levels were lower in the infected than the uninfected children (p = 0.001). As well, the number of children who had IgG levels below that considered protective at one week was more among infected children (8/57, 14%) than among uninfected children (1/54, 2%) (Fishers exact p = 0.03). For ethical reasons all *S*. *mansoni* infected children in group C were treated at one week after catch-up immunisation. At 24 weeks after catch-up immunisation, the IgG levels were not significantly different between children who were infected or uninfected at enrolment. At 24 weeks all the uninfected children (100%) had IgG levels considered protective against measles while 95.7% of the children that were *S mansoni* positive at enrolment had levels considered protective (Fishers exact p = 0.19).

**Table 3:** Association of *S mansoni* infection with antibody levels one and 24 weeks following measles catch-up immunisation.

*S*. *mansoni* status at enrolment	Geometric mean levels of anti-measles IgG mUI/mL (95% CI)	*Adjusted geometric mean ratios (95%CI)
Responses at one week after immunisation		
Uninfected (D, n = 54)	3949 (2661, 5859)	1
Infected (C, n = 57)	1401 (767, 2560)	**0.4 (0.2, 0.7); p = 0.001**
Responses at 24 week after immunisation		
Uninfected (D, n = 51)	2184 (1477, 3230)	1
Infected at enrolment ((A, B, C) n = 138)	2068 (1609, 2659)	1.1 (0.9, 1.5); p = 0.38

* Adjusted for child health days measles immunization history and baseline IgG levels.

### Effect of praziquantel treatment of *S mansoni* on catch-up immunisation anti-measles IgG levels

Having observed an association between *S*. *mansoni* infection and the anti-measles IgG response, we next examined whether praziquantel treatment would have an effect on the antibody levels. This was done by comparing the IgG levels at one week as well as at 24 weeks after immunisation between the three treatment groups as shown in Table 4.

**Table 4:** Effect of praziquantel treatment on antibody levels.

Timing of praziquantel (PZQ) treatment with respect to catch-up measles immunisation (MI)	Geometric means	GM ratios (95% CI)*	% with protective levels	Fisher’s Exact p value
**Levels at one week after the catch-up immunization**				
Not treated (C, n = 57)	1401 (767, 2560)	1	49 (86.0%)	
Treated 2 weeks before MI (A, n = 51)	2233 (1214, 4104)	**2.3 (1.4, 4.5)**	45 (88.2%)	0.955
Treated at the time of MI (B, n = 49)	2339 (1282, 4267)	**1.8 (1.1, 3.5)**	43 (87.8%)	
**Levels at 24 weeks after the catch-up immunization**				
Treated one week after MI (C, n = 50)	2266 (1496, 3431)	1	48(96.0%)	
Treated 2 weeks before MI (A, n = 46)	1858 (1205, 2863)	1.1 (0.7, 1.8)	44(95.7%)	1
Treated at the time of MI (B, n = 42)	2087 (1270, 3430)	1.0 (0.6, 1.6)	40(95.2%)	

*Adjusted for baseline(enrolment) antibody level.

**Fig 3:**
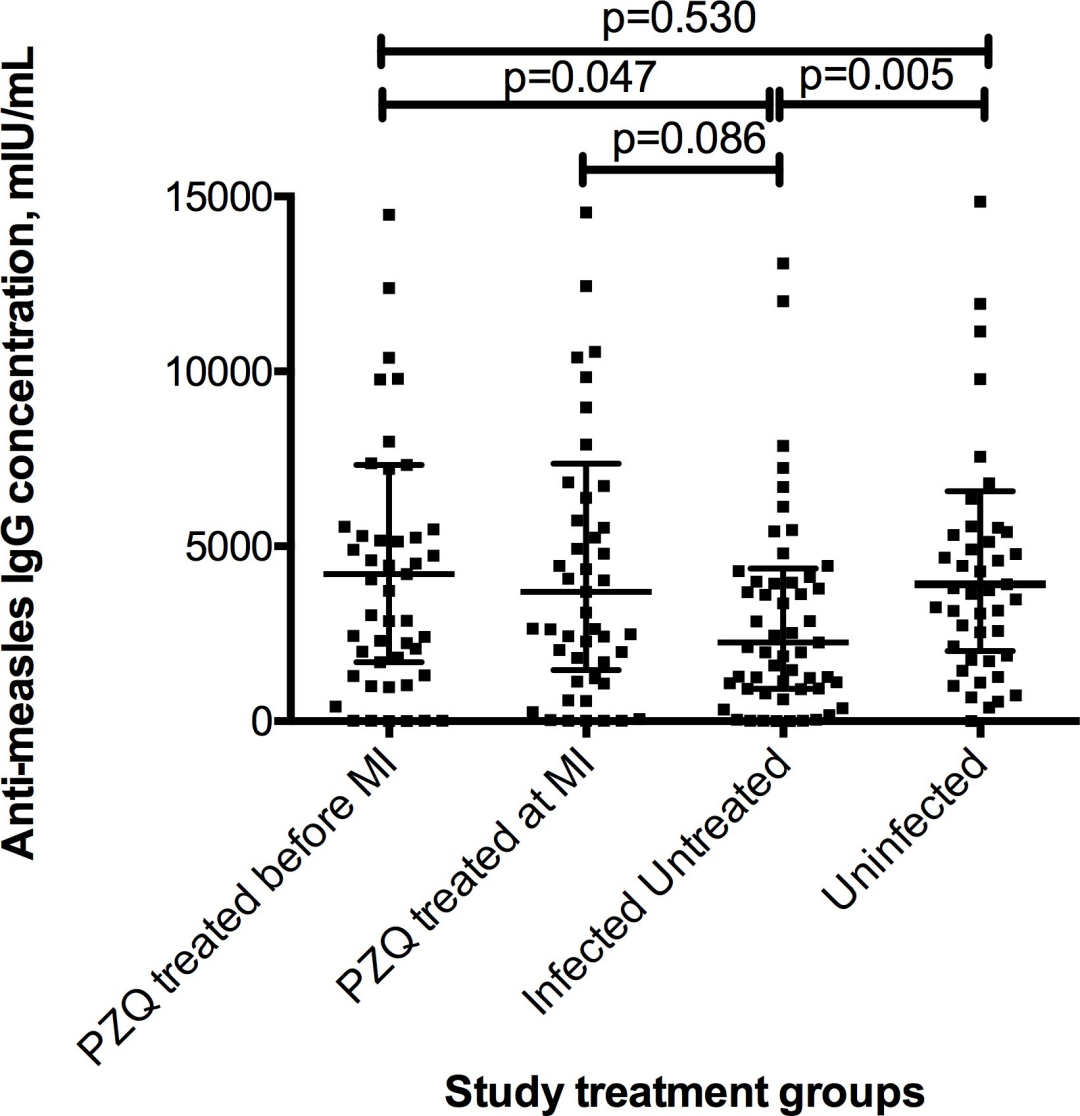
Measles specific IgG levels at one week after catch-up immunization. Error bars shown are median and interquartile whiskers and Wilcoxon rank-sum (Mann-Whitney) test p-values.

At one week after catch-up immunisation, the percentage of children with IgG levels considered protective was over 85% in all the treatment groups and not significantly different between the groups. IgG levels among the children who were treated with praziquantel either before (group A) or at the time of catch-up immunisation (group B) were not significantly different, but were significantly higher than those of children who had not been treated (group C) (Table 4; Fig 3). At week 24 there were no significant differences in the IgG levels between the three treatment groups or between the infected and treated groups and the uninfected group D.

At 24 weeks’ follow-up, 72/138 (52%) of the children who were treated with praziquantel were still *S*. *mansoni* stool positive. Of these children, antibody data was available for 134 participants (70 infected and 64 uninfected). We compared the IgG levels between the children who were stool-positive and those who were stool-negative at the 24-week time point. The IgG levels among children who were infected at enrolment, treated but still *S*. *mansoni* stool positive (GM 1805 (1242, 2624) mIU/ML) were lower than those who remained *S*. *mansoni* stool negative (GM 2379 (1663, 3402) mIU/ML; aGM ratio 0.7 (0.4, 1.0) when adjusted for baseline antibody levels, age and sex. The proportion of children with protective IgG levels was 65/70(93%) among persistently stool positive compared to 63/64(98%) among those who became stool negative (Fishers exact test, p = 0.13).

## Discussion

This study explored the possible effects of *S*. *mansoni* infection on responses to measles catch-up immunisation among three- to five-year-old children, and undertook the first trial to investigate the possible benefits of praziquantel treatment for a vaccine outcome. In an observational analysis we found that *S*. *mansoni* infection was associated with a reduced measles-specific IgG response one week after measles catch-up immunisation and that *S*. *mansoni*-infected children were less likely to develop IgG levels considered protective than their uninfected counterparts. In the trial we found that praziquantel treatment of *S*. *mansoni* infected children resulted in an improved measles-specific IgG response at one week post-immunisation when compared with those who were not treated. This study highlights the possible negative influence of *S*. *mansoni* infection on responses to immunisation and the beneficial effects that treatment with praziquantel could offer.

In our relatively accessible Entebbe population, coverage of measles vaccine (85%), and reported uptake of both 9-month and later child-day doses (over 50%), accorded with the observed baseline proportion with protective antibody levels of greater than 85%. Although measles immunisation in settings such as Uganda may result in protective antibody levels in only three-quarters of the infants immunised [15], a second dose is expected to induce protective levels in 95% of recipients, providing protection for life [33]. This was achieved at 24 weeks for all groups in our study–in which all participants were treated for schistosomiasis before this final endpoint. However, vaccine coverage is lower elsewhere in the country [17, 18]; as well, high prevalence of *S*. *mansoni* among pre-schoolers in endemic communities has recently been reported from Uganda, with treatment of this group advocated [23] but not yet widely practiced. Our results are consistent with the hypothesis that schistosomiasis contributes to impaired measles vaccine efficacy in Uganda (17, 18) although further investigation among the affected communities would be needed to confirm this. While protective levels against measles were indeed attained for most study participants in our study population, the impact of schistosomiasis may be more important in populations with more marginal measles vaccine coverage, and for vaccines that induce more marginal levels of protective immunity.

Helminth infections are associated with induction of an immunologically hyporesponsive state [34] through modulation of innate or adaptive immune functions or both [24, 35, 36]. In particular *S*. *mansoni* has been associated with modulation of antigen-presenting cell function resulting in down-regulation or Th2 polarisation of the adaptive response [37, 38]. Active *Schistosoma* infection is associated with elevated regulatory T cell (Treg) frequencies and suppressed T cell responses to schistosome antigens, and this is alleviated or modified following praziquantel treatment [39–41]. Treatment of schistosomiasis is also associated with changes in circulating cytokine concentrations, such as a decline in circulating IL-10 [42]. The impact of such treatment effects on responses to non-schistosome antigens has been little explored. Our results suggest that there may be an immediate (but perhaps partial) alleviation of suppressive effects on responses to unrelated antigens. As well as removing suppressive effects of active, chronic *Schistosoma* infection, treating schistosomiasis is followed by an immediate immunological “storm” due to the sudden release of adult worm antigen into the host circulation, most marked in heavily infected people [43]: these changes could also impact the response to an unrelated vaccine given concurrently.

The impact of *S*. *mansoni* and its treatment on the response at 24 weeks could not be determined from this study since all infected children had been treated by that time and the immunological effects of treatment, discussed above, may have influenced the later evolution of the measles-specific response. Of interest, the response in children treated after measles immunisation (after sample collection at one week) was similar, by 24 weeks, to levels obtained in children treated before or at immunisation. This suggests that the adverse effects of active *S*. *mansoni* infection on antibody production can be alleviated by post-immunisation treatment. This accords with a study of hepatitis B immunisation in *S*. *japonicum* infected mice: chronically *Schistosoma*-infected mice showed reduced antibody responses and Th2-biased cytokine responses to the hepatitis vaccine antigen, but subsequent treatment with praziquantel resulted in recovery of the antibody response and resolution of the Th2 bias [44]. Our observation that children with persistent *S*. *mansoni* infection had inferior antibody levels at 24 weeks suggests that more effective treatment (perhaps through optimised praziquantel dosing [45], or repeated treatment) and avoidance of *S*. *mansoni* re-infection could deliver further improvements in vaccine response.

We have recently reviewed previous studies on helminths and vaccine responses [46]. While studies in mouse models generally support the hypothesis that chronic helminth infection impairs responses to unrelated vaccines, studies in human populations have produced variable results across a range of helminth species and vaccines. For *S*. *mansoni* several studies in humans indicate an association with reduced antibody responses and, or, Th2 bias [12, 13, 47, 48]. For hepatitis B immunisation, adverse effects may be confined to individuals with hepato-splenic schistosomiasis [49–51] or over-ridden by providing multiple doses [13]. Riner and colleagues recently provided evidence that effects of *S*. *mansoni* co-infection on the response to tetanus immunisation in young adults was greater among individuals with the lowest pre-existing anti-tetanus antibody levels [13]. We recently found no evidence of an effect of concurrent *S*. *mansoni* infection on the response to booster immunisation with the investigational tuberculosis vaccine MVA85A (a live, non-replicating Modified Vaccinia Ankara vectored vaccine, expressing antigen 85A of *Mycobacterium tuberculosis*) among Ugandan adolescents [52]. However, the participants had evidence of substantial prior exposure to mycobacteria (in cellular and antibody responses to mycobacterial antigen, as well as BCG scar) and *S*. *mansoni* infected participants showed a bias to IgG4 antigen 85A specific antibody even before immunisation: perhaps repeated prior mycobacterial exposure dominated the response to this vaccine, over-riding any effects of current *S*. *mansoni* co-infection. This study on measles immunisation was the first to investigate the effects of *Schistosoma* coinfection on the response to a live, replicating attenuated viral vaccine. Such vaccines may be particularly vulnerable to the effects of agents which modify the innate antiviral response and, perhaps, limit vaccine replication [53, 54].

This study was of modest size and sampled participants at a limited number of time points: assessing the antibody response at one week assumed that this would represent the peak of the anamnestic anti-measles response but if some children had never received measles immunisation their peak may have been later. For ethical reasons we did not wish to leave children untreated throughout follow up, limiting our ability to observe longer term effects. To be classified as uninfected children were required to have three samples negative for *Schistosoma* on Kato Katz microscopy and one negative on PCR–a few of them may have had undetected, low intensity infection. The main strength of the study was the trial design, allowing us to determine the effects of treatment, avoiding effects of confounding.

### Conclusion

Our study provides evidence that active *S*. *mansoni* infection may have an adverse effect on measles immunisation, and that treatment of *S*. *mansoni* with praziquantel may provide a benefit which may be diminished by persistent infection or re-infection. The effects of *S*. *mansoni* may be a factor contributing, among several others, to continued cases of measles reported in Uganda and, more widely, to impaired responses to a range of vaccines in tropical settings. There is need to confirm these results, to investigate further the direct impact of helminth infections on responses to childhood, adolescent and adult immunisation, to determine the categories of vaccine affected, and to evaluate the benefits for vaccine response that can be achieved by interventions against helminth infections.
